# Types of Breast Cancer Surgery and Breast Reconstruction

**DOI:** 10.3390/cancers16183212

**Published:** 2024-09-20

**Authors:** Anna Golara, Mateusz Kozłowski, Jerzy Lubikowski, Aneta Cymbaluk-Płoska

**Affiliations:** Department of Reconstructive Surgery and Gynecological Oncology, Pomeranian Medical University in Szczecin, Al. Powstańców Wielkopolskich 72, 70-111 Szczecin, Poland; mtkoozo@gmail.com (M.K.); jerzy.lubikowski@gmail.com (J.L.); aneta.cymbaluk@gmail.com (A.C.-P.)

**Keywords:** mastectomy, breast reconstruction, implants, acellular dermal matrices, autologous reconstruction, robot-assisted surgeries, fat grafting

## Abstract

**Simple Summary:**

Breast cancer is a huge problem in modern medicine, even surpassing breast cancer in women. A very common method of treatment is mastectomy which is, in most cases, followed by breast reconstruction, the course of which changes dynamically. The process is aimed at improving patient satisfaction, minimizing the risk of tumor recurrence, and preventing complications. That is why we decided to look at the trend in the abovementioned procedures. We have described important types of surgical treatment of breast cancer, such as modified radical mastectomy, breast-conserving surgery, contralateral prophylactic mastectomy, and robotic mastectomy. We have also discussed breast reconstruction, focusing on implants, acellular dermal matrix, autologous reconstruction, robotic breast reconstruction, and fat grafting.

**Abstract:**

**Background:** Breast cancer continues to be a significant diagnostic and therapeutic problem. Mastectomy is still a frequently used treatment method, but its form is changing with progress in medicine. **Methods:** We have described important types of surgical treatments for breast cancer, such as modified radical mastectomy, breast-conserving surgery, contralateral prophylactic mastectomy, and robotic mastectomy. Breast reconstruction is also a very important element of treatment because it directly affects the mental state of patients after the procedure. We have also described types of breast reconstruction, such as implants, acellular dermal matrices, autologous reconstruction, robotic breast reconstruction, and fat grafting. **Results:** The aim of our study was to compare available types of surgical treatment for breast cancer and breast reconstruction to help tailor personalized treatment to patients.

## 1. Introduction

**Figure 1:**
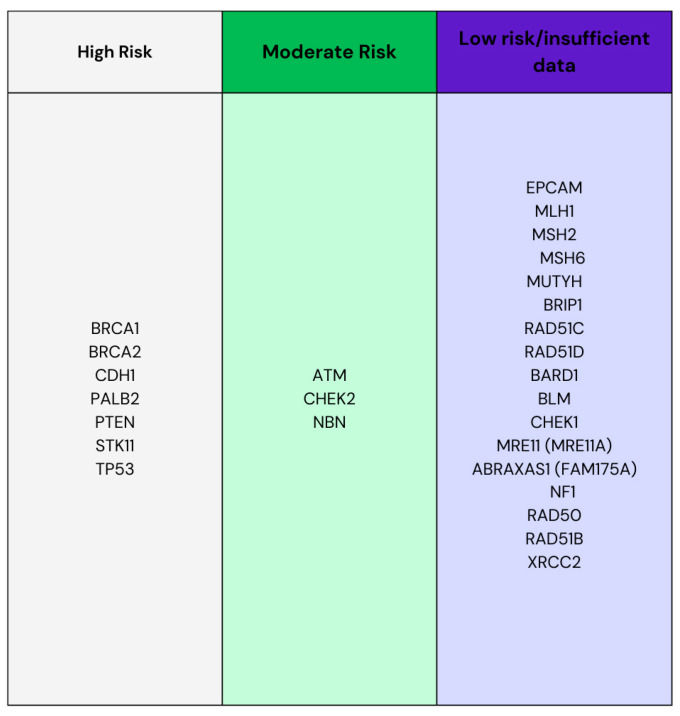
High-, moderate-, and low-risk breast cancer mutations.

Breast cancer is the most frequently diagnosed cancer, with an estimated 2.3 million new cases [1]. Breast cancer can be divided into invasive and non-invasive cancers. The most common diagnosis of breast cancer is invasive ductal carcinoma. Non-invasive breast cancer includes ductal carcinoma in situ and lobular carcinoma in situ [2]. In patients with breast cancer, a high incidence of pathogenic variants of BRCA1 and BRCA2 is observed, as well as other highly mutated genes, such as PALB2, CHEK2, MUTYH, and ATM [3]. In Figure 1, we have presented high-, moderate-, and low-risk breast cancer mutations. Information about the genes whose damage predisposes their carriers to the development of breast cancer facilitates the diagnosis and treatment of this cancer. Treatment for breast cancer depends on its stage. Chemotherapy is used in the preoperative phase, and when tumors express estrogen, progesterone, or ERBB2 receptors, systemic hormonal therapy or immunotherapy may be used in the preoperative phase. The main treatment is surgery, the methods of which are described in our article, while the postoperative phase includes radiotherapy, hormonal therapy, immunotherapy, or chemotherapy. Additionally, if lymph node disease is suspected, a sentinel lymph node biopsy is performed. Breast reconstruction is also an important element of the entire treatment process, which significantly affects the patient’s well-being [4]. The complication rate after breast cancer surgery is low, ranging from 2% to 50%, depending on the method used [5]. The aim of this study was to characterize the types of surgical treatment for breast cancer and breast reconstruction that may be helpful in choosing treatment for a patient, taking into account the patient’s individual needs. This review is based on publications searched in PubMed and Google Scholar. Search terms included (“mastectomy” or “breast removal surgery”) and (“breast reconstructive surgery”). We selected articles whose content presented the current state of knowledge from 2018 to 2024 on types of breast cancer surgery and breast reconstruction.

## 2. Mastectomy

Mastectomy is an operation involving the removal of all or part of the breast gland, often together with the nipple–areola complex. There are four types of mastectomy, radical, modified radical, simple, and partial. We can also see a division between skin-sparing mastectomy and nipple- and areola-sparing mastectomy. Sometimes the procedure is performed to minimize the risk of cancer, in which case we talk about preventive mastectomy. Carriers of the BRCA1 or BRCA2 genetic mutation have an 80 to 85% risk of developing breast cancer [6]. Patients who carry gene mutations for BRCA1, BRCA2, p53, or PTEN or another gene mutation are advised to undergo a preventive bilateral mastectomy [7]. However, because it does not affect patient mortality, prophylactic mastectomy of the contralateral breast is not recommended in average-risk individuals with unilateral disease [8]. In many cases, mastectomy is the main method of treating malignant tumors and is then considered therapeutic. In patients who have a high chance of acquiring breast cancer, prophylactic amputation is performed. This procedure prevents the development of cancer. In a prophylactic mastectomy, both breasts are usually removed. In turn, therapeutic amputation is performed to cure breast cancer, and most often, it is unilateral. However, mastectomy is not the preferred treatment method in the case of metastatic cancer; the oncologist may refer the patient to chemotherapy or hormone therapy. Patients with severe locoregional illness, including large primary tumors (T2 lesions greater than 5 cm) and the involvement of the skin or chest wall, as well as those with inflammatory breast cancer, should consider mastectomy. Patients with multifocal or multifocal disease within the breast should also consider this treatment option. Patients with recurrent breast cancer who have undergone radiation and lumpectomy as prior treatments are also advised to have mastectomy [9]. The cornerstone of care is local surgery, which may be paired with adjuvant or neoadjuvant therapy, such as hormone antagonist medications, radiation, chemotherapy, or a combination of these. The size of the tumor, its location, and the patient’s preferences determine the selection of the appropriate treatment method [10]. A mastectomy is performed on between 30 and 45 percent of all breast cancer patients; roughly 13 percent of these individuals have a contralateral preventive mastectomy performed [11,12].

## 3. Trends in Mastectomy

### 3.1. Modified Radical Mastectomy (MRM)

A common surgical treatment of choice for breast cancer is modified radical mastectomy (MRM), which allows both the removal of the main mass of the tumor and adjacent glandular tissue, in which invasion and multifocality of the process are suspected, as well as the removal of sentinel axillary lymph nodes. Common postoperative complications after MRM include hematoma formation and postoperative wound infection, A common side effect after modified radical mastectomy (MRM) is the formation of serous tissue, which may increase pain and suffering and possibly prolong treatment and morbidity. One treatment method to reduce the incidence of seroma formation may be the intravenous injection of hydrocortisone [13]. Also, flap stabilization with suture after MRM is an alternative treatment method that reduces seroma formation and the amount of drained fluid, enables early removal of the drain, prevents delays in starting adjuvant treatment, is more comfortable for the patient and physician, and is inexpensive [14]. Some studies also suggest combining MRM with neoadjuvant chemotherapy in breast cancer patients. In patients with stage II–III breast cancer, this combination reduces the incidence of side effects, effectively inhibits serum tumor markers (STMs), and improves quality of life (QOL) [15]. Xie et al. examined the impact of such therapy on the course of treatment in 80 patients. Compared to the control group, patients in the study sample had considerably shorter hospital stays and operating times, as well as lower bleeding volumes and higher success rates from their treatments. In addition, compared to the control group, the treatment group exhibited a significantly lower incidence of complications and a superior quality of life. Additionally, neoadjuvant treatment was demonstrated by Cox regression analysis to be an independent factor impacting the patients’ progression-free survival [16].

Additionally, it has been proposed that in patients undergoing modified radical mastectomy (MRM), the erector spinae plane (ESP) block will offer superior postoperative pain relief compared to the serratus anterior muscle (SAM) block [17,18]. Without significantly impairing hemodynamic stability, ESF provides analgesic efficacy during surgery and the postpartum phase that is comparable to thoracic epidural analgesia. Since neuraxial blocks are linked with a higher rate of complications than paraspinal blocks, ESB appears to be a viable substitute technique for providing efficient pain management during mastectomy surgeries [19].

Because MRM necessitates both an ipsilateral axillary lymph node dissection and the removal of the whole breast, it is frequently carried out under general anesthesia (ALND). Nonetheless, it has been demonstrated that individuals with significant comorbidities have a higher risk when undergoing general anesthesia. In Pandya et al.’s case study, a 78-year-old man with heart failure and other metabolic diseases was found to have invasive ductal breast cancer. Following a tumescent, minimal-pain local anesthetic injection, the man underwent MRM and ALND. The patient underwent a safe, effective operation with little discomfort [20]. A significant amount of research supports the finding that MRM can be transferred to the outpatient setting for appropriate patients [21].

### 3.2. Breast-Conserving Surgery

Patients treated with oncoplastic breast-conserving surgery (OBCS) may experience longer operative times; however, they have significantly less intraoperative bleeding, postoperative drainage, and hospitalization times compared to patients treated with MRM. Patients treated with OBCS also demonstrate higher subjective satisfaction and quality of life, as well as better objective outcomes. In terms of the incidence of postoperative complications and recurrence rates, there is no significant difference between breast-conserving surgery and modified radical mastectomy [22,23].

Senoga et al. compared the quality of life of patients with early breast cancer (ECC) at least 1 year after BCT or MRM. The study included 42 patients after BCT and 39 after MRM. Patients who underwent BCT had a better overall quality of life than those who underwent MRM (*p* = 0.0149). A multivariate analysis showed that five years after surgery, the level of education and presence of diabetes significantly (*p* < 0.05) influenced the quality of life of these patients [24].

### 3.3. Contralateral Prophylactic Mastectomy

The incidence of contralateral prophylactic mastectomy (CPM) for unilateral breast cancer (UBC) continues to increase despite the lack of clear survival benefit [25], except in patients at the highest risk of developing contralateral breast cancer [26]. CPM decisions are also frequently observed in patients with ATM, CHEK2, and PALB2, which are the three most common moderate-risk breast cancer genes [27]. Currently, there are no established guidelines regarding qualification for CPM; however, it is recommended that the procedure be “dissuaded” in women at an average risk of contralateral breast cancer and considered in all women at the highest risk of contralateral breast cancer, including women with a BRCA1 or BRCA2 mutation. Unfortunately, CPM is associated with an increased number of postoperative complications and a longer recovery period, which may lead to delays in adjuvant cancer treatment [28], although some studies contradict this finding [29].

Different trends are observed among different age, racial, and ethnic groups. According to the literature, between 2004 and 2017, women of color and women over the age of 65 were less likely to have CPM or reconstruction than their white counterparts. Therefore, further research is needed to understand the factors that influence decision-making [30].

Many satisfaction studies have been conducted after contralateral prophylactic mastectomy among BRCA mutation carriers and noncarriers. Myers et al. examined 149 patients with the BRCA mutation and 842 patients who were not carriers. Satisfaction and well-being were similar in BRCA carriers and noncarriers treated with CPM. Compared with noncarriers, BRCA carriers experienced greater declines in breast satisfaction and well-being 6 months after CPM [31]. It is also a good idea to involve partners in pre- and postoperative counseling, which may have the benefit of alleviating confusion regarding expected oncological and emotional outcomes associated with CPM [32].

### 3.4. Robot-Assisted Mastectomy

Nipple-sparing mastectomy (NSM) is used to achieve better cosmetic results in patients with early breast cancer. This procedure requires a higher level of skill and places a greater physical burden on operators than mastectomy.

This treatment is currently being used in selected patients who meet specific indications of RNSM, i.e., robot-assisted NSM. However, there are four concerns regarding RNSM, including increased costs, oncologic outcomes, experience and skill levels, and standardization. However, the robotic system provides greater precision and accuracy, helping to remove breast tissue more effectively. The great advantages of this procedure are smaller scars, less blood loss, lower rates of surgical complications [33], and better patient-reported quality of life [34,35,36].

Because breast cancer is very often diagnosed in patients at an early stage, there has been increased interest in methods that could provide better cosmetic results. The minimally invasive procedure, the newest trend in surgery, is being followed by the robotic surgery system, which is growing in popularity. The da Vinci SP Surgical System is one of the most recent iterations of the robot (Intuitive Surgical) [37]. The benefits of using the da Vinci^®^ robotic surgical system (Intuitive Surgical, Sunnyvale, CA, USA) include a small 3D camera and lighting for excellent visualization, Endowrist robotic instruments offering a greater range of motion, and the ability for the surgeon to work in a more ergonomic position, i.e., sitting at the console [38].

Go et al. evaluated clinicopathological features, surgical results, and postoperative complications in 81 retrospectively (70 patients), using the Mann–Whitney U test to ascertain the viability of robotic-assisted nipple-sparing mastectomy (RNSM) using the da Vinci single-port (SP) system with a small incision, concealed in the arm. The median age of the patients was 42 years. Eleven patients had bilateral RNSM, with a median initial skin incision size of 40 mm. Fifteen patients had deep inferior epigastric perforator flaps (DIEP), and fifty-four underwent immediate repair with direct implant implantation. Six (7.5%) individuals experienced postoperative complications classified as III using Clavien–Dindo. Larger breasts with more severe ptosis were present in patients with the DIEP flap; nonetheless, no grade III problems occurred. Therefore, regardless of breast size or degree of ptosis, the researchers propose that RNSM using the SP system can be employed for therapeutic mastectomy [39]. However, surgeons must have experience and knowledge of the system to maximize its benefits and minimize risks or complications. Unfortunately, further research is needed to clarify the oncological safety and cost-effectiveness of RNSM.

## 4. Trends in Breast Reconstruction

Breast reconstruction aims to improve the quality of life of women with breast cancer. The aim is to treat the distorted shape of the breast and improve the therapeutic effect of oncological surgery while adjusting the symmetrical appearance of the breast. In times of medical staff, individual treatment should be selected for each patient, paying attention to problems related to implants, psychological burden, and rehabilitation costs.

Prosthetic implants, or implant-based breast reconstruction (IBR), and free autologous tissue transfer, or autologous breast reconstruction (ABR), are the two most popular techniques for breast reconstruction. Autologous breast reconstruction involves the use of flaps (tissues taken from the donor area and used to reconstruct the breast area after appropriate shaping). This technique allows you to obtain a natural appearance of the breast with features similar to the original and can be used in an irradiated field. The deep inferior epigastric perforator (DIEP) and transverse rectus abdominis musculocutaneous flaps (MS-TRAM) are the two most often utilized free flaps [40]. Mastectomy is becoming an increasingly conservative procedure, which allows for improved aesthetic reconstruction results, especially when an implant is used. The prosthesis can be placed in front of the muscle (prepectoral reconstruction) if the appropriate thickness of the mastectomy flap is maintained, or behind the muscle (submuscular reconstruction). With prepectoral reconstruction, postoperative recovery is faster, and the postoperative appearance is more natural than with submuscular reconstruction [41].

A special type of patient is obese patients, who constitute quite a large percentage of the total patients and have a higher failure rate in the case of implant-based breast reconstruction, especially immediate reconstruction. Flap techniques or delayed implant reconstruction may be warranted in this population [42]. Although implant reconstruction after mastectomy is still a commonly used method of reconstruction, autologous breast reconstruction has increased in popularity in recent years [43].

### 4.1. Implants

**Figure 2:**
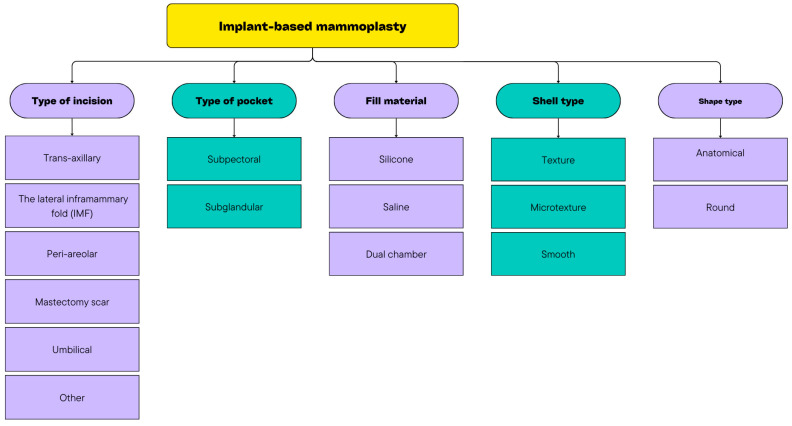
Characteristics of implant-based mammoplasty.

Currently, we have several types of implants to choose from in breast reconstruction, as follows: cohesive gel implants, highly cohesive gel (i.e., gummy bear) implants, saline implants, and structured saline implants with different surfaces and shapes from which to choose [44] [Figure 2].

The lightweight B-Lite^®^ breast implant (G&G Biotechnology Ltd., Haifa, Israel) is also available on the market. It enables the surgeon to achieve the patient’s desired breast size and shape while lowering the risk of long-term breast tissue deformation, as well as damage to the integrity and stability of the breast tissue. However, avoiding tissue damage and deformation, and ultimately reoperation, improves both patient safety and satisfaction [45].

The B-Lite breast implant weighs less than conventional silicone implants by about 30% while keeping the same size, shape, and functionality. This means that the breast tissues are not under as much pressure and are able to retain their stability and integrity over time, which lowers the risk of weight-related complications and the need for repeated operations [46]. Tessmann et al. conducted a retrospective study that analyzed 48 patients (38 implants in each group) who underwent implant-based breast reconstruction using B-Lite^®^ implants or conventional breast implants. The postoperative observation consisted of a clinical examination and a survey using the BREAST-Q questionnaire to assess the postoperative quality of life. In many respects, the quality of life in both groups appeared similar; however, patients who received B-Lite^®^ implants had significantly better results in terms of sensitivity in the surgical area and scar formation. Unfortunately, B-Lite^®^ implants were perceived by patients as more uncomfortable [47].

### 4.2. ADM

ADM is an acellular dermal matrix consisting of a structurally integrated complex of basement membrane and extracellular matrix. It is used to wrap the implant during surgery in the prepectoral plane. To realize the potential of ADM, proper patient selection, surgical placement, and postoperative management are important because these factors contribute to the proper integration of the matrix with surrounding tissues. ADM acts like a graft, so it requires hard and healthy tissue to establish itself. Therefore, for the procedure to be successful, the following three key steps must be followed: appropriate patient selection, conservative and gentle intraoperative technique, and meticulous postoperative management [48]. Acellular dermal matrix (ADM) has been observed to reduce the fibrotic response seen in the foreign body response (FBR), but the mechanism is poorly understood [49].

The human acellular dermal matrix has become an increasingly used adjunct to traditional submuscular tissue expander/implant breast reconstruction, but its use has been correlated with an increased likelihood of complications, such as infection and flap necrosis [50].

Acellular dermal matrices (ADM) are commonly used in prepectoral breast reconstruction, although they are expensive and associated with the potential for infection and seroma. Prepectoral reconstruction with and without ADM was compared by comparing 515 reconstructions from four studies. The vast majority of cases involve nipple-sparing mastectomy and reconstruction using a tissue expander. The meta-analysis showed no significant difference in the incidence of complications between the ADM and non-ADM cohorts. Short-term complications were reconstructive failure (1.2% in the ADM cohort and 2.8% in the non-ADM cohort), seroma (1.2% and 8.3%, respectively), hematoma (1.2% and 2.1%), infection (4.7% and 4.2%), and postmastectomy flap ischemia and/or necrosis (2.4% and 5.2%). Long-term complications include heaving (3.3% in non-ADM and 5.1% in non-ADM cohorts) and capsular contracture (6.8% and 3.4%, respectively) [51].

Additionally, Rodriguez et al. [52] examined satisfaction levels, aesthetic outcomes, and postoperative complications in patients with breast cancer who underwent rapid prosthetic reconstruction with or without biological mesh following skin- or nipple-sparing surgical surgery. Patients with cT2 tumors that did not respond to primary systemic treatment, ductal carcinoma in situ that indicated mastectomy, and patients with multifocal breast cancer were included in the study. Patients who were older than 75 years old, had inflammatory carcinoma, or had serious circulatory issues were excluded. Patients who underwent prosthesis-assisted reconstruction made up the control group, while patients who underwent prosthesis-assisted reconstruction utilizing biological acellular porcine dermal mesh (Strattice^TM^, Strattice, Warsaw, Poland) made up the research group. BREAST-Q was used to evaluate the outcome. The study included a total of 51 patients, and the control group included 38 patients. A total of 5.9% of patients in the experimental group and 24.3% of patients in the control group (*p* = 0.030) experienced implant loss or removal. A total of 4.8% of the study group’s patients and 7.3% of the control group’s patients had infections; (*p* = 1.00). A total of 12.2% of patients in the study group and 21.6% of patients in the control group (*p* = 0.367) had skin necrosis. A total of 12.2% of the research group and 8.1% of the control group individuals had serum (*p* = 0.514). In terms of “satisfaction with breasts after surgery” (*p* = 0.026), “sexual well-being after intervention” (*p* = 0.010), and “satisfaction with the information received” (*p* = 0.049), the BREAST-Q questionnaire compares the two groups. The findings point to a statistically significant reduction in implant loss in female biomesh recipients and increased patient satisfaction in Strattice^TM^-reconstructed patients.

ADM may also be associated with red breast syndrome (RBS), which is an inflammatory event usually manifesting as skin erythema at the site where the ADM is surgically implanted. Therefore, further research is needed into the prevention and treatment of RBS to improve patient outcomes. RBS caused by patient hypersensitivity to certain ADMs has been documented in the literature and improved by switching to an alternative brand of ADM [53]. There are two types of ways to use ADM, including the freeze-dried type and the pre-hydrated type. The hydrated type of ADM was designed to be softer than the freeze-dried type. A retrospective chart review study of 78 patients (using 26 freeze-dried type, 52 pre-hydrated type, MegaDerm; L&C BIO, Seongnam, Republic of Korea) was conducted to compare the two types of ADM. In the aesthetic assessment, the shape and symmetry of the breasts assessed by both the doctor and the patients were better in the pre-hydrated type group, which was related to the nature of the texture. In terms of complications and the amount of drainage, the outcome did not differ significantly between the two groups [54]. When comparing two standard acellular dermal matrix companies, AlloDerm SELECT Ready To Use and DermACELL, a higher percentage of sera was found in AlloDerm-treated breasts (30.91%) compared to DermACELL-treated breasts (14.55%, *p* < 0.05), and a statistically significant difference was observed between the inclusion rates of AlloDerm (93.4%) and DermACELL (99.8%, *p* < 0.05). Despite this, both products had a 94.55% success rate in terms of reconstructive outcomes [55].

It is also noted that obesity, a history of smoking, and insulin-dependent diabetes are independent risk factors for superficial wound infections in patients with ADM [56].

Radiotherapy is an integral part of breast cancer treatment, but it significantly increases the incidence of overall complications in breast reconstruction. To reduce the incidence of complications in irradiated fields, an acellular dermal matrix (ADM) is used. A retrospective analysis of a single-center experience with ADM-assisted implant-based reconstruction or revision surgery for the treatment of capsular contracture in irradiated breasts was performed. The study group was divided into the following four groups based on previous surgical history: group A (previous quadrantectomy), group B (previous mastectomy and expander reconstruction), group C (previous mastectomy and implant reconstruction), and group D (previous quadrantectomy followed by mastectomy and reconstruction implant). A total of 84 patients were identified and underwent a total of 86 irradiations of breasts reconstructed with implant and ADM. A total of 12 reconstructive failures were observed, with the highest failure rates in group B (16.6%) and group D (15.38%). A total of 24.4% of general complications were recorded, of which the most common complication was infection. Group B had the highest rate of complications, both major and minor, with 16.6% experiencing each. Before breast reconstruction with ADM, Baker’s score was between 3 and 4, with an average of 3.25. At the 2-year follow-up, the Baker score ranged between 1 and 4, with an average of 1.9. The aesthetic result was very satisfactory in 72.1% of cases, moderately satisfactory in 8.1%, and unsatisfactory in 5.81%, and in 13.9%, the result was not assessable due to reconstructive failure, with the worst aesthetic result recorded in group B. A significant reduction in capsular contracture was observed in revision surgery despite moderately high complication rates in previous quarantectomy and radiotherapy. It has been suggested that breast reconstruction with implant and ADM is not a primary surgical indication in the setting of prior irradiation but may be considered a valid alternative with a reasonable safety profile for use in selected cases [57]. However, adjuvant chemotherapy may be an interesting therapeutic option. Scardina et al. investigated that prepectoral immediate prosthetic breast reconstruction (PP-IPBR) after NAC neoadjuvant chemotherapy is a safe, reliable, and effective alternative to traditional submuscular immediate prosthetic breast reconstruction (SM-IPBR). PP-IPBR gives excellent aesthetic and oncological results, is also easy to perform, shortens operation time, and minimizes complications related to PPM manipulation [58].

Woussen et al. assessed the impact of skin matrices on quality of life and complications. To assess the quality of life in the case-control study, they used the following two surveys: BREAST-Q V2.0© and QuickDASH to compare the responses between the “Matrix+” and “Matrix-” groups. Seventeen IBRs with matrices (23.6%) and 55 IBRs without matrices (76.4%) were analyzed. “Matrix+” patients had a better quality of life in terms of sexual well-being (*p* = 0.038), significantly lower QuickDASH (*p* < 0.01), better satisfaction with breasts (*p* = 0.016), and better satisfaction with implants (*p* < 0.01). The probability of subsequent serious complications was higher in the matrix group (*p* = 0.04). This suggests a higher quality of life, sexual well-being, and satisfaction with breasts and implants thanks to the use of matrices; however, due to the higher number of serious late complications, immediate prosthetic reconstruction of the breast with a matrix should be considered after analyzing the comorbidities and viability of the skin flaps of each patient [59].

Currently, work is being performed to support the operator’s work with robotic devices, as well as in breast surgery. We evaluated the results of surgical breast reconstruction directly on implants using an acellular dermal matrix after a nipple-sparing mastectomy assisted with a robotic device (Da Vinci Xi). The study included thirty-nine cases, including seven bilateral cases (46 breasts in total). The average operating time for each reconstruction of the prepectoral breast mound using the direct-to-implant technique was 126.55 min. Overall satisfaction with the robot was rated as better than with the conventional reconstruction method using BREAST-Q. In seven cases (15.2%), there was a serious infection, and in three (6.6%), there was a complete loss of nipples. Severe complications requiring surgical removal of breast implants were observed in four breasts (8.7%). Two cases involved the coexistence of infection and skin necrosis, while in one case, the skin flap underwent a congestive phase on the postoperative day (POD), which required additional surgery to change the expander. Other complications required conservative treatment or minor revision. Further studies are needed to evaluate oncological outcomes [60].

Acellular dermal matrices (ADM) are biologically modified tissues that act as an immunologically neutral scaffold in breast reconstruction. Many problems arise in the imaging of ADM in follow-up examinations of patients after reconstruction and in distinguishing normal conformation from residual or recurrent disease, which is why radiologists’ training in this area is so important [61].

Implant-based breast reconstruction is commonly performed using a human acellular dermal matrix. Broyles et al. compared the two most commonly used human acellular dermal matrices in immediate implant-based breast reconstruction after mastectomy at seven clinical centers. They divided the patients into two groups. Patients from group A received FlexHD Pagile (MTF Biologics, Berlin, Germany), and patients from group B received AlloDerm RTU (AlloDerm, North Chicago, IL, USA). The study found no statistical difference in overall array complications between groups A and B. Obesity and prepectoral array placement were independently associated with a higher risk of overall array complications [62].

Skin autografts are being used more and more often. Initially, in women who had excess breast tissue, they were used to create tissue expanders and implants in the lower field during breast reconstruction. Then, the abdominal skin autografts were collected. Subsequent studies have evaluated the use of mesh skin autografts. Skin autografts can be easily harvested during mastectomy from abdominal incisions or excess breast tissue. When comparing ADM and cutaneous autografts histologically, autografts have significantly greater neovascularization, which potentially reduces the incidence of complications. In addition, skin autografts, especially meshed dermal autografts, enable significant coverage of the prosthesis, which acts as a barrier in the event of wound dehiscence, which protects the prosthesis and allows for local wound healing. However, the results in terms of aesthetic effects and capsular contracture are comparable in both methods. Dermal autografts are also more financially advantageous compared to ADMs [63].

### 4.3. Autologous Reconstruction

Delayed or immediate breast reconstruction with a transverse rectus abdominis (TRAM) flap is possible. Since the tissue is a component of the patient’s body, capsular contracture and inflammatory reactions—common issues linked to breast implants—are avoided. Assessing the patient is the first step in determining if they are a good candidate for this operation. The breast deformity and the amount required to fix it should be the doctor’s main concerns. Matching the tissue volume of the muscle flap to the volume of the other breast is an important element in achieving aesthetic success. The reconstructive procedure uses the abdominal pannus. A thinner patient with larger breasts is a more challenging clinical case because she may need a contralateral breast reduction or the implantation of a rectus abdominis muscle (TRAM) flap implant. In contrast, a thicker abdominal scale for smaller breast reconstruction frequently necessitates secondary symmetry revision. The primary benefit of the treatment for the patient is the restored breast’s resemblance to the original [64]. A higher quality of life is observed in patients after autologous breast reconstruction than in patients after implant-based reconstruction. There was no evidence that the procedure was unsafe [65]. We have described the various autologous tissues used in reconstruction in Table 1.

### 4.4. Robot-Assisted Breast Reconstruction

Work using robots is also being improved in reconstruction. Investigators compared the results of robotic-assisted breast reconstruction with a latissimus dorsi flap after partial mastectomy with those of conventional and endoscopic-assisted techniques in 57 Korean patients, taking into account surgical outcomes and patient satisfaction. A total of 20 patients underwent conventional reconstruction, and 17 and 20 patients underwent endoscopic and robotic procedures, respectively. There was no statistically significant difference between the three methods in postoperative opioid analgesic dosage (*p* = 0.459), hospitalization period (*p* = 0.225), and mean total amount of donor site drainage during hospitalization (*p* = 0.175). Taking into account patient satisfaction after the procedure, particularly with regard to the scar at the donor site, the conventional method showed a significantly lower result than the other two techniques [74].

Jeon et al. conducted a study that included sixteen patients (16 breasts) who underwent mastectomy with direct-on-implant (DTI) reconstruction using the da Vinci XiTM device (Intuitive Surgical Corp., Sunnyvale, CA, USA), using the anterior plane tenting method with an ADM. Two patients had skin-sparing mastectomies, and fourteen patients had nipple-sparing mastectomies. The oncology team operated for an average of 194.7 min, whereas the plastic surgery team operated for 80.8 min. Postoperative drainage averaged 943.6 mL, and two patients experienced minor complications. During surgery, you can access areas that are not easily visible by making a small incision about 4.5 cm long. Because the ADM is smaller, it is simple to build and enlarge the implant pocket beneath it. Robotic surgery can also be used to reconstruct the inframammary fold (IMF), and it is very simple to manage delayed pouch hemorrhage [75].

There are also attempts to use the robotic system with da Vinci SP for capsulectomy, which reduces the risk of joint capsule contracture and thus contributes to better aesthetic results but may also be associated with complications, such as damage to the axillary structures or chest wall, and overlapping skin extravasation may occur on it. The robotic system can minimize the risk of injury because it has freely moving arms and clear, magnified three-dimensional vision for total capsulectomy [76].

Robotic-assisted breast reconstruction is associated with less skin necrosis and better patient-reported outcomes (higher sexual well-being score and higher physical well-being score) than the conventional option according to the BREAST-Q questionnaire [77]. Therefore, robotic surgery may be a good solution for mastectomy and breast reconstruction.

### 4.5. Fat Grafting

Fat grafting is used at various stages of the reconstructive process. Fat injection can be used as a complement to implant-based and autologous breast reconstruction, but also as a primary reconstructive option in eligible patients. It allows you to achieve the look and feel of a naturally supple breast and alleviates some of the negative effects associated with mastectomies [78]. The oncological safety of fat grafting for breast reconstruction is questioned, although there is no clinical evidence to suggest an increased risk of recurrence or development of new cancer [79,80]. Theoretically, adipose tissue grafts contain progenitor cells and immunomodulatory cytokines that may induce vascularization, tumor progression, or recurrence at this site, so further research is necessary [81].

### 4.6. No Reconstruction

A relatively new movement is the Going Flat movement, a group for women who opt out of breast reconstruction after mastectomy. Research conducted by Baker et al. [82] assessing motivation and satisfaction with surgical results in such patients suggests that the majority of women who decide not to undergo reconstruction are satisfied with the results. Therefore, plastic surgeons should ensure that patients are fully informed about reconstruction, ensuring a comprehensive understanding of its risks, benefits, and alternatives and supporting them in their decisions.

## 5. Conclusions

1. Advances in breast reconstruction have led to increased patient satisfaction, quality of life, and aesthetic results while maintaining oncological safety; however, the choice of breast reconstruction depends on the type of mastectomy, necessary radiation, individual risk factors, and patient preferences.
2. The surgeon’s decision to use ADM in prepectoral breast reconstruction should be made after carefully analyzing each patient case and taking into account patients’ hypersensitivity to some ADMs.
3. Many breast reconstruction methods are currently available, so comparative studies are necessary to match patients to appropriate methods.
4. The learning curve for robotic surgery appears to be manageable and can be overcome with appropriate training and practice.

## Figures and Tables

**Table 1:** Types of autologous breast reconstruction.

Tissue Origin	Tissue Used	References
Abdomen	Transverse rectus abdominis myocutaneous flap (TRAM) The deep inferior epigastric artery perforator flap (DIEP) Superficial inferior epigastric artery flap (SIEA)	[64,66,67]
Back	Latissimus dorsi flap (for the back muscle that is used)	[68]
Buttocks	Superior gluteal artery perforator flap for breast reconstruction with autologous tissue (S-GAP flap) Inferior gluteal artery perforator (I-GAP flap)	[69,70]
Thighs	Gracilis-based flaps - Transverse upper gracilis flap (TUG flap) - Vertical upper gracilis flap (VUG flap) - Diagonal upper gracilis flap (DUG flap) Posterior thigh-based profunda artery perforator (PAP flap)	[71,72,73]
